# Use of SGLT2 Inhibitors in Patients with Chronic Kidney Disease and Urinary Tract Infection: Is There a Need for Concern?

**DOI:** 10.3390/jcm14207302

**Published:** 2025-10-16

**Authors:** Demet Yavuz, Havva Yasemin Çinpolat, Ayşe Kevser Demir, Nezaket Kadı, Öznur Kal, İremcan Şahin, Sevil Alkan, Göksenin Ünlügüzel Üstün, Nihal Aydemir

**Affiliations:** 1 Department of Nephrology, Faculty of Medicine, Samsun University, 55090 Samsun, Türkiye; 2 Department of Medical Biochemistry, Faculty of Medicine, Çanakkale Onsekiz Mart University, 17400 Çanakkale, Türkiye; 3 Department of Internal Medicine, Faculty of Medicine, Samsun University, 55090 Samsun, Türkiye; 4 Department of Internal Medicine, Samsun Training and Research Hospital, 55090 Samsun, Türkiye; 5 Department of Nephrology, Başkent University Konya Practice and Research Hospital, 42080 Konya, Türkiye; 6 Department of Infectious Diseases and Clinical Microbiology, Faculty of Medicine, Çanakkale Onsekiz Mart University, 17400 Çanakkale, Türkiye; 7 Department of Medical Biochemistry, Samsun Training and Research Hospital, 55090 Samsun, Türkiye

**Keywords:** chronic kidney disease, diabetes, sodium-glucose cotransporter-2 inhibitors, proteinuria, urinary tract infection

## Abstract

**Objective**: This study aimed to investigate urinary tract infections (UTIs) and associated risk factors in patients with type 2 diabetes mellitus and chronic kidney disease (CKD), with or without treatment with sodium-glucose cotransporter-2 inhibitors (SGLT2i). **Methods**: We retrospectively analyzed diabetic CKD patients with available urine culture results. Patients were divided into two groups: those receiving SGLT2i therapy and those not receiving it. The groups were compared retrospectively with respect to the development of urinary tract infection at 12-month follow-up, using clinical and laboratory results. **Results**: A total of 151 patients with T2DM were included, with a median age of 70 years (range: 61–76), and 84 (56%) were female. Among them, 91 (60%) patients were treated with SGLT2i. BMI, plasma glucose levels, and the urine protein/creatinine ratio were significantly lower in the SGLT2i group (*p* = 0.002, *p* = 0.049, and *p* < 0.001, respectively), while serum urea and creatinine levels were significantly higher (*p* = 0.048 and *p* = 0.028, respectively). A total of 59 patients (39.1%) had positive urine cultures, 32 of whom (35.2%) were using SGLT2i. There was no statistically significant difference in UTI incidence between SGLT2i users and non-users (*p* = 0.298). Among patients with positive cultures, Escherichia coli was the most common pathogen, identified in 52.5% (n = 31) of cases. Patients with positive urine cultures were older (*p* = 0.005), and 39 (66%) were female (*p* = 0.038). According to logistic regression analysis, advanced age and female sex were identified as independent risk factors for UTI (*p* = 0.037; Odds Ratio = 2.172, 95% CI: 1.048–4.502 and *p* = 0.033; Odds Ratio = 2.169, 95% CI: 1.065–4.415, respectively). **Conclusions**: In diabetic patients with CKD, the use of SGLT2i reduces proteinuria without increasing the risk of urinary tract infections. Advanced age and female sex are independent risk factors for UTI.

## 1. Introduction

Chronic kidney disease (CKD) is a common and progressive disease associated with significant mortality and morbidity. Diabetes is a common cause of CKD, and both diabetes and CKD increase the risk of cardiovascular disease (CVD), the leading cause of death in individuals with CKD. The first two approaches to slowing the progression of diabetic kidney disease are a renin-angiotensin system (RAS) inhibitor and a sodium-glucose cotransporter 2 inhibitor (SGLT2i). Combining these agents provides greater cardiorenal risk reduction than either agent alone. Preventing the progression of CKD is crucial for reducing CVD risk in individuals with CKD and diabetes [1].

In CKD, the glomerular filtration rate (GFR) progressively declines, and reducing albuminuria—a major risk factor for CKD progression—is of great importance [2]. In patients with diabetic nephropathy and significant albuminuria, the use of renin-angiotensin system (RAS) inhibitors [1,3], sodium-glucose cotransporter-2 inhibitors (SGLT2i) [1,4], and the non-steroidal mineralocorticoid receptor antagonist finerenone [1,5] has been shown to slow the progression to end-stage renal disease. Delaying CKD progression is crucial to protect patients from the increased morbidity and mortality associated with renal replacement therapies such as dialysis [6].

DM is a globally prevalent disease with an increasing incidence, affecting multiple organ systems. Due to its chronic nature, it leads to numerous complications, primarily nephropathy. DM negatively affects both humoral and cellular immunity, primarily by impairing the antioxidant defense system, thereby increasing susceptibility to various infections—particularly urinary tract infections (UTIs) [7]. Poorly controlled hypertension and diabetes, acute kidney injury (AKI), worsening cardiac function, exposure to nephrotoxic agents, and hypotensive events are recognized as key factors contributing to the progression of chronic kidney disease (CKD). Moreover, infectious episodes in CKD patients have been linked to an increased risk of disease progression and a faster transition to end-stage renal disease [8].

In addition to inducing glucosuria, SGLT2i have been shown—even in non-diabetic patients—to lower blood glucose, promote weight loss through glucose-induced osmotic diuresis, and reduce proteinuria by restoring impaired tubuloglomerular feedback mechanisms [9]. Despite their well-established renoprotective effects, concerns have been raised that the glucose-rich urinary environment associated with SGLT2i use may promote bacterial growth and increase the risk of UTIs [10,11]. However, recent studies have found no statistically significant relationship between SGLT2i use and increased UTI risk [12]. A Canadian study also emphasized that a history of UTI within the past 60 days did not increase the risk of UTI among SGLT2i users [13] and that the risk was comparable to other drug classes [14].

Beyond diabetic patients, SGLT2 inhibitors are now recognized as first-line agents in the treatment of heart failure and diabetic nephropathy and are even considered safe for use in immunosuppressed kidney transplant recipients [15].

This study aimed to evaluate whether the use of SGLT2i in pre-dialysis diabetic CKD patients increases the risk of UTIs by comparing clinical and laboratory parameters between users and non-users.

## 2. Materials and Methods

### 2.1. Study Design

This retrospective study was conducted at Samsun University, Samsun Training and Research Hospital, and was approved by the Non-Interventional Clinical Research Ethics Committee of Samsun University, Samsun, Türkiye (Approval No: GOKAEK 2024/10/9).

This study included type 2 diabetic patients who were followed at the nephrology outpatient clinic between January 2023 and January 2024. Patients with type 2 DM diagnosed and treated in accordance with ADA guidelines and using SGLT2i regularly were included [16]. All patients underwent clinical and laboratory evaluation. Data were collected from the medical records of patients over the age of 18 with early-stage CKD who had been using renin-angiotensin system (RAS) blockers—including angiotensin-converting enzyme inhibitors (ACEi) or angiotensin II receptor blockers (ARBs)—for at least six months, and who were either using or not using SGLT-2i agents such as empagliflozin or dapagliflozin.

Exclusion criteria included: patients under 18 years of age; non-diabetic individuals; those who discontinued SGLT2i during follow-up; patients with incomplete data; individuals with type 1 diabetes; pregnant women; patients with a history of rhabdomyolysis; those undergoing renal replacement therapy; individuals using nephrotoxic drugs or radiocontrast agents; and patients with benign prostatic hyperplasia, obstructive uropathy, bladder dysfunction or malignancy, or active genital infections. Data from hospitalized patients with active infections were excluded; instead, only follow-up data (e.g., eGFR, proteinuria, HbA1c, fasting blood glucose (FBG) obtained after a clinically stable period of at least one month were included in the analysis. A history of prior UTI was not considered an exclusion criterion, as in some patients, SGLT2i therapy was initiated shortly after resolution of the previous infection. There was no use of urinary catheters among our patients.

Genital infections were evaluated based on patient-reported symptoms including increased sensitivity, redness, swelling, changes in color, odor, and increased discharge in the labia majora or minora, though no routine physical examination of the genital area was conducted. Diagnoses such as vaginitis and vulvitis in women, and balanitis, orchitis, epididymitis, and balanoposthitis in men were classified as genital infections taking into account the electronic digital recorded physician notes.

### 2.2. Data Collection

Data collection included demographic characteristics (age, sex, BMI), history of hypertension, hospitalization status, length of hospital stay (in days), and laboratory findings: complete blood count, CRP, FBG, HbA1c, and fully automated urinalysis including pyuria, hematuria, bacteriuria, glucosuria, proteinuria, and nitrite positivity. Incomplete medical records were supplemented using the national health database (e-Nabız).

Midstream urine samples were obtained for culture. Data on urine culture positivity, isolated pathogens, and antibiotic treatment were recorded. EUCAST (European Committee on Antimicrobial Susceptibility Testing) criteria were used in our hospital laboratory for urine culture determination. A urine culture was considered positive if bacterial growth was ≥10^5^ CFU/mL. UTI was diagnosed based on both clinical symptoms and positive culture results. Patients with UTI received appropriate antibiotic treatment for 7–10 days. In hospitalized patients, repeat urine cultures were obtained post-treatment to confirm eradication. No cases of pyelonephritis, urosepsis, or mortality were observed.

Prescription data were electronically tracked, including the frequency of all oral antidiabetic and insulin prescriptions. In Türkiye, medications are prescribed for up to three months and can be monitored via the e-Nabız system, allowing us to determine prescription frequency for SGLT2i and other agents during follow-up.

Patients using and not using SGLT2i were compared in terms of UTI incidence, BMI, FBG, HbA1c, protein/creatinine ratio, glucosuria, and proteinuria. Venous blood testing was not routinely performed but available results from outpatient visits were included. Euglycemic diabetic ketoacidosis diagnosis was based on clinician notes within the electronic medical record system.

Complete blood counts were analyzed using an automated hematology analyzer (Sysmex XN-1000, Sysmen Corporation, Kobe, Japan). Urinalysis was performed using the BT URICELL 1280 + 1600 fully automated analyzer (Biotecnica Intruments S.p.A., Rome, Italy). Fasting blood glucose was measured with the AU5800 Series Chemistry Analyzer (Beckman Coulter Inc., Brea, CA, USA) using the glucose oxidase method. HbA1c was measured by high-performance liquid chromatography and reported in both International Federation of Clinical Chemistry units (mmol/mol) and National Glycohemoglobin Standardization Program units (%).

### 2.3. Statistical Analysis

Statistical analyses were performed using SPSS version 17.0 (IBM Corporation, New York, NY, USA). The distribution of data was assessed using the Kolmogorov–Smirnov test. Normally distributed variables are presented as mean ± standard deviation and compared using the independent samples *t*-test. Non-normally distributed variables are presented as median (1st quartile–3rd quartile) and compared using the Mann–Whitney U test. Categorical variables are expressed as number (percentage) and compared using the Chi-square test or Fisher’s exact test, as appropriate. Logistic regression analysis was conducted using the Enter method, including all covariates, to identify independent risk factors for UTI. A *p*-value of less than 0.05 was considered statistically significant.

## 3. Results

A total of 151 patients with type 2 DM were included in this study. The median age was 70 years (range: 61–76), and 84 patients (56%) were female. Among them, 91 (60%) were receiving SGLT2i. The median ages of patients using and not using SGLT2i were 69 (range: 64–76) and 71 (range: 58–78) years, respectively, with females comprising 61.5% (n = 56) and 46.7% (n = 28) of each group. BMI, FBG, and urine protein/creatinine ratio were significantly lower in the SGLT2i group (*p* = 0.002, *p* = 0.049, and *p* < 0.001, respectively). Conversely, serum urea and creatinine levels were significantly higher among SGLT2i users (*p* = 0.048 and *p* = 0.028, respectively). Glucosuria was more prevalent in the SGLT2i group, while proteinuria was more frequent in non-users (*p* < 0.001 and *p* = 0.025, respectively). Detailed patient characteristics are summarized in Table 1. Notably, eGFR remained stable in the SGLT2i user group after one year compared to baseline.

Among the patients, 59 (39.1%) had positive urine cultures, with 32 (35.2%) of these patients being users of SGLT-2 inhibitors. There was no statistically significant difference between SGLT2i users and non-users regarding the presence of urinary tract infections (UTIs) or hospitalization due to UTI (*p* = 0.298). Of the bacteria isolated from urine cultures, Escherichia coli accounted for 31 cases (52.5%), and Klebsiella pneumoniae for 12 cases (20.3%). Less frequently isolated microorganisms included *Candida* spp., *Pseudomonas aeruginosa*, *Citrobacter koseri*, *Enterococcus faecalis*, *Myroides* spp., *Serratia odorifera*, *Streptococcus agalactiae*, and *Enterobacter cloacae* (Table 1).

Patients with UTIs were significantly older than those without infections (*p* = 0.005), and 39 (66%) of them were female (*p* = 0.038). Additionally, HbA1c, FBG, and serum urea levels were significantly higher in patients with UTIs (*p* < 0.001, *p* < 0.001, and *p* = 0.024, respectively), whereas eGFR was significantly lower (*p* = 0.033) (Table 2).

Logistic regression analysis was conducted using the Enter method with continuous covariates. The overall model was statistically significant (Omnibus χ^2^ = 77.911, *p* < 0.001) and demonstrated good fit (Hosmer-Lemeshow *p* = 0.484). Female sex (Odds Ratio = 3.02, 95% CI: 1.15–7.92, *p* = 0.025) and higher HbA1c levels (Odds Ratio = 3.21, 95% CI: 2.10–4.92, *p* < 0.001) were found to be independent predictors of urinary tract infection. Although age approached statistical significance (*p* = 0.076), it did not reach the conventional threshold (Table 3).

## 4. Discussion

SGLT2i have revolutionized diabetes treatment by providing significant benefits not only for diabetic patients but also for those with cardiovascular disease. By reducing proteinuria and lowering intraglomerular pressure, these agents modulate the tubuloglomerular feedback mechanism, thereby contributing to renal protection and serving as an almost miraculous therapeutic option for patients with kidney disease [9]. However, concerns have been raised regarding the increased risk of genital and UTIs associated with SGLT2i treatment [9,17,18]. Given the immunocompromised status of patients with CKD, an elevated incidence of UTIs in this population might be expected. In this study, treatment of hyperglycemia with SGLT2i in diabetic patients with early-stage CKD was associated with reduced proteinuria without an increased incidence of UTIs.

CKD affects approximately 8% to 16% of the global population [19], with diabetes and hypertension being the most common etiologies. Clinical evaluation of CKD involves staging based on GFR and albuminuria, followed by comprehensive risk factor assessment aimed at preventing progression. Optimal treatment strategies include controlling blood pressure and glycemia, as well as reducing albuminuria through agents such as ACE inhibitors or ARBs [19]. The use of SGLT2i in nephrology practice has gained acceptance over time as accumulating evidence supports their renal benefits and safety profile. This gradual adoption is expected, as CKD patients are often excluded from drug trials due to safety concerns and included only after safety is established. Our findings are consistent with this trend, demonstrating that SGLT2i, increasingly used for their cardioprotective and renoprotective effects even in non-diabetic patients, may not increase the risk of UTIs in diabetic CKD patients and are safe to use in this population.

As is widely recognized, SGLT2i exert cardioprotective and renoprotective effects independent of their glucose-lowering properties. In diabetic CKD patients, canagliflozin treatment has been shown to reduce CKD progression risk by 30% [20]. Similarly, dapagliflozin has demonstrated renoprotective effects by reducing proteinuria levels [21]. Notably, one major clinical trial involving SGLT2i was terminated early due to clear benefits observed in CKD patients [22]. In the DAPA-CKD study, administration of 10 mg dapagliflozin significantly reduced the risk of CKD progression in patients without type 2 DM [3]. Furthermore, analysis revealed that dapagliflozin reduced eGFR decline, the risk of end-stage kidney disease, and renal or cardiovascular mortality regardless of diabetic status. In our study, patients treated with SGLT2i exhibited stable eGFR at one year and significant proteinuria reduction. The absence of eGFR decline is likely attributable to decreased proteinuria, suggesting a protective effect against CKD progression.

SGLT2i act by blocking glucose reabsorption in proximal tubules via SGLT2 channels, leading to glucosuria [23]. Glucosuria has been proposed as a key mechanism predisposing patients to UTIs during SGLT-2i therapy [24]. In type 2 diabetes, overexpression of SGLT2 channels elevates the renal threshold for glucose excretion [25]. Although some studies have linked the glucosuric effects of SGLT2i to increased UTI risk [26], others have not found a significant association [27,28,29]. Our results align with the latter, showing that these effects may not increase UTI risk despite 62.6% of SGLT2i users exhibiting glucosuria compared to 18.3% of non-users. Interestingly, previous research in patients with normal renal function found lower glucosuria prevalence but higher UTI incidence among SGLT2i users, suggesting that glucosuria alone may not fully explain UTI development [30]. Furthermore, the increased incidence of urinary tract infections (UTIs) in individuals with diabetes may be due to differences in host responses in diabetic patients, differences in infecting microbial strains, or a combination of both. While the exact mechanisms remain partially elucidated, a number of possible hypotheses have been proposed to elucidate the link between diabetes and UTIs, including altered growth conditions resulting from glycosuria and diabetes-associated neuropathy and altered pathogen-host interactions resulting from diabetes. Although more than half of the patients in our study had glycosuria, glucosuria alone is not sufficient to prevent UTIs; it may be due to the host susceptibility factors and alternative mechanisms mentioned above [12].

No significant differences in sex or age distribution were observed between SGLT2i users and non-users; however, UTIs predominantly affected female patients. Female sex was identified as a independent UTI risk factor, likely due to factors such as reduced hygiene and the shorter female urethra. Additionally, patients with UTIs demonstrated poorer glycemic control (higher FBG and HbA1c), elevated serum urea, and lower eGFR, suggesting that hyperglycemia and dehydration may exacerbate UTI risk and impair renal function. Additionally, no cases of diabetic ketoacidosis or hypoglycemia were reported among SGLT2i users.

SGLT2i are recommended as first-line agents for cardiovascular risk reduction regardless of glycemic control. Mori et al. reported that cardioprotective effects diminished with decreasing BMI and were negligible in patients with BMI < 25 kg/m^2^, highlighting the importance of BMI consideration in therapy initiation [31]. Consistent with prior studies, SGLT2i therapy was associated with weight loss, modest glycemic improvements, and low adverse event rates [32]. Our findings showed lower BMI and serum glucose in SGLT2i users compared to non-users; however, no relationship between BMI and UTI incidence was observed. Elevated urea and creatinine levels in the SGLT2i group may be explained by hypotension, urinary sodium loss, and suboptimal hydration.

In diabetic patients, RAS inhibitors delay progression from microalbuminuria to macroalbuminuria and reduce cardiovascular mortality [33]. Beyond hemodynamic effects, SGLT2i confer renal protection via favorable modulation of energy metabolism, inflammation, and fibrosis pathways [34]. Our study supports these renoprotective effects, as SGLT2i users exhibited significantly lower proteinuria.

Limitations of this study include its retrospective design and the lack of assessment of genital infections, which might influence UTI risk. Additionally, eGFR measurements prior to SGLT2i initiation were not available, limiting insight into early renal hemodynamic changes. A 12-month follow-up may not be sufficient to fully determine the long-term risk of UTI in the diabetic chronic renal failure population; further studies are needed where patients can be followed for longer periods. Additionally, in our study, UTIs were not classified as mild, recurrent, or severe (e.g., pyelonephritis). Clinically classifying UTIs into three groups would have provided a better understanding of the effects of SGLT2 inhibitor use on UTIs. Different SGLT2 inhibitors, such as Empagliflozin or Dapagliflozin, may have different effects on the risk of UTIs. Due to the limited number of cases in our study, we were unable to distinguish between drug subtypes. Planning a study with a larger sample size and drug subtypes could shed light on understanding UTIs in patients with end-stage renal disease. Another limitation is that immunosuppressant or corticosteroid use and history of recurrent UTI were not recorded due to the retrospective nature of this study. Moreover, our dataset lacked the exact date of UTI diagnosis and follow-up duration per patient. Therefore, we conducted logistic regression analysis based on the presence or absence of UTI during the observation period. Cox regression model could not be used in our study. We acknowledge this as another limitation.

In conclusion, SGLT2i use in diabetic CKD patients reduces proteinuria and stabilizes renal function without increasing UTI risk or hospitalization related to UTIs. Given their benefits in reducing major renal and cardiovascular events and slowing CKD progression, concerns over UTI risk should not deter SGLT2i use in this population.

## Figures and Tables

**Table 1:** Comparison of Sociodemographic Characteristics and Laboratory Results of Patients Using and Not Using Sodium-Glucose Cotransporter-2 Inhibitors.

Parameters	SGLT2i (+) n = 91	SGLT2i (−) n = 60	*p*-Value
Age (year)	69 (64–76)	71 (58–78)	0.630
Sex (female)	56 (61.5%)	28 (46.7%)	0.072
BMI (kg/m^2^)	24 (21–26)	25 (22–29)	0.002 *
Hospitalization (yes)	13 (14%)	13 (22%)	0.355
Duration of hospitalization (day)	7 (4–13)	10 (5–12)	0.254
Systolic blood pressure (mmHg)	125 (120–130)	130 (120–136)	0.259
Diastolic blood pressure (mmHg)	70 (70–75)	70 (70–80)	0.284
CKD period (year)	4 (1–5)	3 (2–7)	0.547
HT period (year)	7 (4–10)	7 (4–9)	0.348
Glycated hemoglobin (%)	7.0 (6.0–8.0)	7.4 (6.5–10.3)	0.755
FBG (mg/dL)	114 (92–142)	139 (111–165)	0.049 *
Urea (mg/dL)	71 (51–90)	60 (48–91)	0.048 *
Creatinine (mg/dL)	1.88 ± 0.47	1.76 ± 0.47	0.028 *
eGFR CKD-EPI	37 (30–45)	40 (32–51)	0.193
Uric acid (mg/dL)	6.2 (5.3–7.9)	7.3 (5.4–8.6)	0.842
Total protein (g/L)	66.4 ± 7.1	67.9 ± 6.3	0.595
Albumin (g/L)	36.3 ± 5.7	36.1 ± 4.9	0.393
Calcium (mg/dL)	9.08 ± 0.78	9.01 ± 0.82	0.361
Phosphorus (mg/dL)	4.1 (3.7–4.8)	4.2 (3.7–4.6)	0.765
Parathyroid hormone (pg/mL)	83 (51–204)	139 (88–202)	0.964
25-hydroxy vitamin D (ng/mL)	16 (7–27)	14 (8–24)	0.869
C reactive protein (mg/L)	6.8 (3.3–44.0)	14.7 (3.2–62.8)	0.402
Leukocyte count (10^3^/µL)	7.50 (6.45–9.38)	8.19 (6.09–10.02)	0.658
Hemoglobin (g/dL)	10.6 ± 0.7	10.4 ± 0.8	0.580
Platelet count (10^3^/µL)	261 ± 88	258 ± 81	0.475
Protein creatinine ratio	0.7 (0.35–1.3)	1.75 (1.15–2.68)	<0.001 *
Urine analysis			
Nitrite (positive)	3 (3.3%)	3 (5.0%)	0.682
Bacteriuria (positive)	15 (16.5%)	15 (25.4%)	0.259
Pyuria (positive)	41 (45.1%)	31 (52.5%)	0.370
Hematuria (positive)	29 (32.6%)	19 (32.2%)	0.961
Glucosuria (positive)	57 (62.6%)	11 (18.3%)	<0.001 *
Ketonuria (positive)	1 (1.1%)	3 (5%)	0.301
Proteinuria (positive)	47 (51.6%)	42 (70.0%)	0.025 *
Density	1.013 (1.010–1.019)	1.012 (1.009–1.017)	0.234
Urine culture, UTI (positive)	32 (35.2%)	27 (46.6%)	0.298
*Escherichia coli*	18 (56.3%)	13 (48.1%)	
*Klebsiella pneumonia*	6 (18.8%)	6 (21.4%)	
*Candida*	2 (6.3%)	2 (7.1%)	
*Pseudomonas aeroginosa*	1 (3.1%)	2 (7.1%)	
*Citrobacter coseri*	0	1 (3.6%)	
*Enterococcus faecalis*	1 (3.1%)	1 (3.6%)	
*Myroides* spp.	0	1 (3.6%)	
*Serratia odorifera*	1 (3.1%)	0	
*Streptococcus agalactia*	1 (3.1%)	0	
*Enterobacter kloaka*	2 (6.3%)	1 (3.6%)	

* *p* < 0.05 was considered as statistically significant. Data was presented as number (percentage), mean ± standard deviation or median (1st quartile–3rd quartile).

**Table 2:** Comparison of Sociodemographic Characteristics and Laboratory Results of Patients With and Without Urinary Tract Infections.

Parameters	UTI (−) (n = 92)	UTI (+) (n = 59)	*p*-Value
Age (year)	66 ± 13	72 ± 10	0.005
Sex (female)	45 (49%)	39 (66%)	0.038
Glycated hemoglobin (%)	6.6 (6.0–7.1)	8.5 (7.2–10.0)	<0.001
FBG (mg/dL)	109 (91–124)	165 (154–184)	<0.001
Urea (mg/dL)	60 (48–78)	65 (55–90)	0.024
eGFR CKD-EPI	39 (31–50)	35 (30–40)	0.033
Hemoglobin (g/dL)	10.7 ± 0.6	10.4 ± 0.7	0.009

*p* < 0.05 was considered as statistically significant. Data was presented as number (percentage), mean ± standard deviation or median (1st quartile–3rd quartile).

**Table 3:** Logistic Regression Analysis of Factors Affecting Urinary Tract Infection in Diabetic Patients with Chronic Kidney Disease.

Variables	B	S.E.	O.R.	95% CI for O.R.	*p* Value
Age	0.040	0.023	1.041	0.996–1.088	0.076
Sex (Female)	1.104	0.493	3.016	1.149–7.923	0.025
SGLT-2i (+)	0.765	0.496	2.149	0.812–5.688	0.123
Glycated hemoglobin	1.167	0.217	3.213	2.100–4.918	<0.001
Urea	0.017	0.012	1.017	0.994–1.042	0.152
Hemoglobin	−0.119	0.326	0.888	0.469–1.682	0.715

B; regression coefficient, S.E.; standard error, O.R.; Odds ratio, CI; confidence interval, FBG; fasting blood glucose.

## Data Availability

The data supporting the findings of this study are available from the corresponding author upon reasonable request.

## Abbreviations

CVD	cardiovascular disease
ACE	angiotensin converting enzyme
ARBs	angiotensin II receptor blockers
BMI	body mass index
CKD	chronic kidney disease
DM	diabetes mellitus
FBG	fasting blood glucose
eGFR	estimated glomerular filtration rate
HbA1c	glycated hemoglobin A1c
RAS	renin-angiotensin system
ROC	Receiving Operating Curve
SGLT2i	sodium-glucose cotransporter-2 inhibitors
UTIs	urinary tract infections
EUCAST	European Committee on Antimicrobial Susceptibility Testing
